# Research on a Machine Learning-Based Method for Assessing the Safety State of Historic Buildings

**DOI:** 10.1155/2022/1405139

**Published:** 2022-08-23

**Authors:** Xiao-Hong Peng, Zi-Hao Zhang

**Affiliations:** ^1^School of Architecture, Anhui Science and Technology University, Bengbu, Anhui 233000, China; ^2^School of Architecture, South China University of Technology, Guangzhou, Guangdong 510000, China

## Abstract

Historic and protected buildings are increasingly valued due to their valuable historical and cultural value. The assessment of the safety state of historic buildings has received more attention. Emerging machine learning algorithms, with their excellent computational performance, provide new ideas and new means to solve practical problems in various fields. Therefore, this paper proposes a method for assessing the safety state of historic buildings based on machine learning techniques. Firstly, based on the analysis of the characteristics of historical buildings and common security problems, the application of wireless sensor networks to the security monitoring of historical buildings is proposed in order to improve the automation of monitoring. Then, in order to improve the accuracy of the assessment, a combination of kernel canonical correlation analysis (KCCA) and support vector machine (SVM) is used to establish the security monitoring model. The experimental results show that by choosing a suitable KCCA function, the redundant features of the data can be reduced while the comprehensiveness of the building structure identification features can be retained, thus effectively improving the prediction accuracy of the SVM. The KCCA-SVM model can accurately predict the physical quantities such as relative structural displacement of historical buildings with good reliability.

## 1. Introduction

Outstanding historical buildings are either the former residences of great men and celebrities or traditional buildings with unique architectural styles and cultural connotations. These buildings are a distillation of the history of a city or region and document the architectural culture of the area. Historic and protected buildings are a proud urban landscape and a rare and valuable cultural heritage [1–4]. Therefore, we need to strengthen the protection of historic buildings through relevant laws and regulations, and at the same time adopt better technical means to protect the safety of historic buildings.

The first step should be to ensure the safety of the structure from the point of view of structural safety. On the one hand, due to the great age of these historic buildings, the performance of the building materials has deteriorated severely. There are some historical buildings that have undergone many alterations, and their use has changed. In addition, most of these historical buildings designed and built decades or centuries ago have not been considered for earthquake resistance. On the other hand, the rapid development of modern cities, the emergence of high-rise buildings, and urban metros have caused varying degrees of impact on these old buildings in the vicinity, all of which are potential safety hazards. Structural safety monitoring technology has a prominent role to play in monitoring and maintaining the safety of buildings [5–9]. Therefore, installing structural monitoring systems on historic buildings to predict their safety state is an effective technical tool.

**Figure 1:**
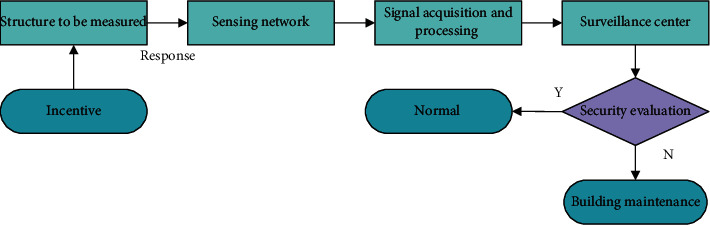
Working principle of the security monitoring system.

Safety monitoring techniques differ from traditional nondestructive evaluation (NDE) techniques, which usually measure the physical state of a building structure directly [10–13]. The results of NDE evaluations depend heavily on the resolution and accuracy of the measurement equipment. Monitoring techniques, on the other hand, predict the state of a structure based on changes in measurements at different times at the same location. Historical data are therefore crucial, and the accuracy of the predictions is dependent on the sensors and interpretation algorithms. Advanced structural safety monitoring technology is a real-time automated system that requires no human intervention and is capable of automatically assessing the safety state of a building via a local area network or remote center. It is generally accepted that a structural safety monitoring system should consist of 2 main components [14–16]: (1) a sensor system, including the selection of sensing elements and the arrangement scheme of the sensor network in the structure, and (2) a data acquisition and analysis system. The working principle of the safety monitoring system is shown in Figure 1.

It is more important to take preventive measures to protect historic buildings than to restore them in a state of imminent destruction. Existing security monitoring systems are less automated and less real time, which makes it difficult to meet the actual needs. The use of wireless sensor networks [17–19] for security monitoring of historic buildings is a more advanced technology than current methods of building security monitoring. As shown in Figure 1, one of the key steps in a security monitoring system is the security state assessment [20–22]. However, it is often difficult to accurately describe the nonlinear relationships between complex data in the security state assessment of historic buildings. With the emergence of various machine learning algorithms in recent years, machine learning algorithms are used to solve this problem and good computational results can be achieved. Based on monitoring data from wireless sensor networks, machine learning algorithms are combined with traditional security monitoring theory to fully exploit the information inherent in the monitoring data, thereby improving the accuracy of the security posture assessment of historic buildings.

The rapid development of machine learning algorithms has led to computers being able to better mimic human learning behavior. Machine learning algorithms continuously acquire new knowledge through autonomous learning and achieve self-renewal for solving new problems [23–26]. Currently, many machine learning algorithms are widely used with their good search capability and fast computing speed, providing new means to solve various problems in multiple fields. Similarly, machine learning algorithms have been widely used in traditional construction engineering, such as the application of Bayesian learning, genetic algorithms, and neural networks. Goodfellow et al. [27] used fuzzy mathematical methods to identify horizontal and vertical displacements and displacement distributions of buildings. Hejazi et al. [28] investigated the fuzzy relationship between various influencing factors and the displacement of offshore buildings. Di Napoli et al. [29] proposed the use of CNNs in the fitting and prediction analysis of building landslide monitoring data.

Compared to neural network-based algorithms, support vector machine (SVM) has obvious advantages in solving small samples, nonlinear, and high-dimensional data processing [30]. Duarte and Wainer [31] used least squares support vector machines for building deformation prediction, and the designed model has good feasibility, validity, and high prediction accuracy. Jain et al. [32] used support vector machines for building safety early warning models with high model accuracy. Tamilarasi and Prabu [33] used particle swarm algorithms to optimize support vector machines in order to perform inverse analysis of building safety model parameters. However, the SVM-based monitoring model will fully extract a large number of nonlinear features and noise, which inevitably increases the complexity of the model operations and affects the accuracy of the prediction.

Kernel canonical correlation analysis (KCCA) is an important method for multidimensional feature correlation analysis [34], in which variable features of different dimensions are correlated in order to remove redundant features. KCCA can reduce the dimensionality of variables while reducing the interference of noise, which helps to reduce the computational complexity of the monitoring model and improve the accuracy of the final prediction.

Therefore, this paper attempts to combine the two approaches to build a KCCA-SVM-based security monitoring model for historic buildings. Firstly, wireless sensor networks are applied to the security monitoring of historic buildings in order to improve the automation of monitoring. Secondly, KCCA technique is used for feature correlation analysis to reduce the dimensionality of a large amount of nonlinear data. Then, SVM's advantages in handling nonlinear and high-dimensional data are fully utilized to predict a wide range of physical quantities of historic buildings, improving the accuracy of security state assessment. The aim of this study is to automatically assess the security state of historic buildings using a KCCA-SVM-based security monitoring model. The proposed method helps to achieve automated monitoring of historic buildings over time to ensure the safety of these buildings.

The main innovations and contributions of this paper include the following.

1. Application of wireless sensor networks to the security monitoring of historic buildings in order to improve the automation of monitoring.
2. A historical building safety monitoring model based on KCCA-SVM is developed to address the problems of variable dimensionality and noise interference in the traditional monitoring model based on SVM. KCCA-SVM can improve the final prediction accuracy while reducing the operational complexity of the monitoring model.

The rest of the paper is organized as follows: In Section 2, the characteristics of historic buildings and common safety issues are studied in detail, while Section 3 provides the principles associated with the KCCA-SVM model.  Section 4 provides the security monitoring model for historic buildings based on KCCA-SVM.  Section 5 provides the project examples. Finally, the paper is concluded in Section 6.

## 2. Characteristics of Historic Buildings and Common Safety Issues

### 2.1. Characteristics of Historic Buildings

As history has continued to develop, a rich and diverse range of architectural types has developed in each region and is also a visual representation of the extent of economic and cultural development. To this day, the surviving historic buildings show distinctive characteristics in terms of three aspects: architecture, architectural style, and supporting facilities.

#### 2.1.1. Building Structure

Due to the limitations of the technical means, the historical buildings formed a structure mainly made of wood. At the same time, modern architectural structures have been added in modern times to form a complete system. Due to the direct exposure of wooden structures to the air, historic buildings are highly susceptible to spontaneous combustion in the event of prolonged heat or lightning strikes. Historic buildings are also susceptible to ignition when other open flames are present.

#### 2.1.2. Architectural Style

**Figure 2:**
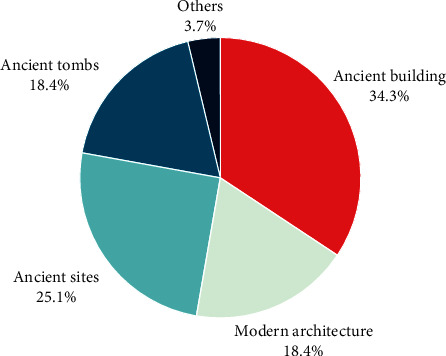
Percentage of types of immovable cultural objects.

According to the different architectural styles, historical buildings can be roughly divided into traditional ancient buildings and recent historical buildings. Traditional ancient buildings are mostly residential buildings, which are characterized by their layout according to the axis. Modern historical buildings have consciously retained the appearance of traditional buildings and absorbed some Western architectural styles. However, both types of historic buildings perform poorly in terms of fire resistance and seismic performance. According to the Third National Cultural Relics Census in 2011, there are 766,722 immovable cultural relics in China, of which 34.42% are in the category of ancient buildings and 18.45% are in the category of modern historical buildings, as shown in Figure 2.

#### 2.1.3. Supporting Facilities

Firstly, due to the lack of funds for renovation and the difficulty of retrofitting, the electrical installations of many historic buildings are seriously deteriorating. Secondly, many historic buildings cannot be equipped with natural gas pipelines and therefore have to use liquefied petroleum as a domestic energy source, which undoubtedly poses a huge safety hazard. Finally, due to the age of the buildings, some of them lack effective structural support and are less able to withstand earthquakes.

### 2.2. Common Safety Issues

Geological and meteorological hazards do not occur as frequently as fires, but when they do, the damage to historic buildings is often very significant. Geological hazards are mainly classified into transient and slow-onset geological hazards. Earthquakes, landslides, and ground subsidence are classified as transient geological hazards. These “natural” disasters are devastating to historic buildings once they have been affected. Soil erosion and ground subsidence, on the other hand, are slow-onset disasters. These hazards are characterized by the cumulative damage that they cause to historic buildings. Once they reach a critical point, they can damage the building itself.

### 2.3. Wireless Sensor Network-Based Security Monitoring of Historic Buildings

Currently, traditional bus-based monitoring systems are used for the safety monitoring of historic buildings. Bus-based monitoring systems can control a variety of disaster detectors and fire-fighting equipment. However, the biggest drawback of the bus system is the wired connection, which is an inherent problem of traditional technology. In wireless monitoring systems, data are transmitted wirelessly. No wires are required to connect the sensors to the collection units, which greatly reduces the amount of labor required for on-site installation and minimizes damage to historic buildings. The wireless sensing unit in the wireless monitoring system realizes the acquisition and wireless transmission of signals, is small in size, consumes little power, and can be battery powered.

**Figure 3:**
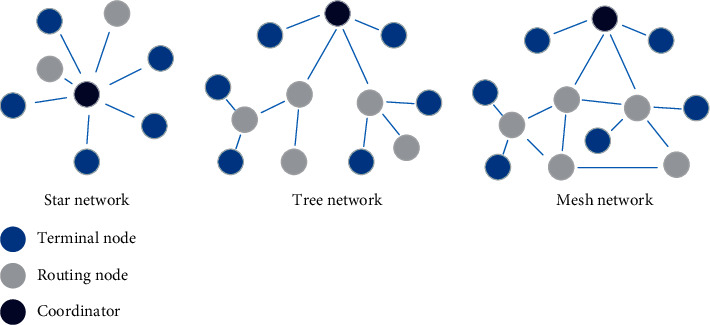
Three common topologies for wireless sensor networks.

A wireless sensor network is a multi-hop, self-organizing system that uses wireless communication. A wireless sensor network consists of multiple discrete sensor nodes randomly deployed in a monitoring area. As a new environmental monitoring technology, wireless sensor network has the advantages of real time, large range, automation, and all-weather. The use of wireless sensor networks for historical building monitoring can improve the automation of historical building safety monitoring and enhance the real-time nature of monitoring. The three common topologies of wireless sensor networks are shown in Figure 3.

**Figure 4:**
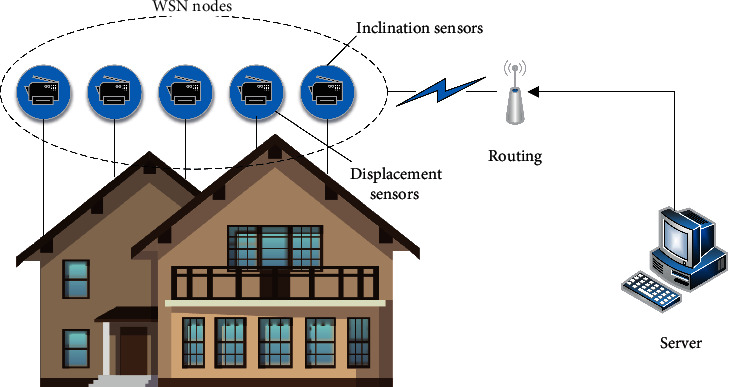
Wireless sensor network-based safety monitoring system for historic buildings.

For historical building safety monitoring, the environmental data to be monitored include physical quantities such as the settlement of the house and the tilt of the walls. Therefore, inclination sensors, displacement sensors, and pressure sensors need to be deployed at relevant locations. In this paper, a wireless sensor network with a tree topology is used to implement a historical building safety monitoring system. The nodes of the wireless sensor network transmit the collected physical quantities to the routing node in a multi-hop manner, and the routing node sends the data to the monitoring computer. The structure of the wireless sensor network-based historical building safety monitoring system is shown in Figure 4.

**Figure 5:**
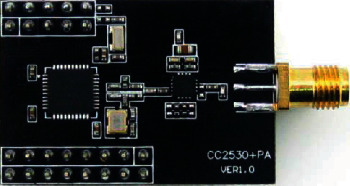
Wireless sensor network nodes.

The main components of a wireless sensor network node include a microprocessor module, a wireless transceiver module, a power supply module, a debugging interface module, and a sensor module. The first four modules are common to the node, while the node has different sensor modules for different functions. The microprocessor module is the core of the sensor node, which is mainly responsible for collecting and processing local data. The sensor node controls the wireless transceiver module to complete tasks such as data transmission. The microcontroller of the node is an STM32F103 chip from STMicroelectronics. The wireless RF chip is an Atmel AT86RF231 chip. The wireless RF chip supports the IEEE802.15.4 standard, works in the 2.4 GHz band, and also supports communication protocols such as RF4CE, Zigbee, and 6LoWPAN. The wireless sensor network node is shown in Figure 5.

## 3. Principles Associated with the KCCA-SVM Model

### 3.1. Typical Correlation Analysis

**Figure 6:**
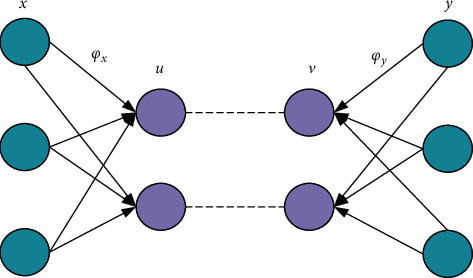
CCA schematic diagram.

Typical correlation analysis (CCA) is commonly used to quantify the correlation between two multidimensional data [35], and its main structure is illustrated in Figure 6.

Let a set of mean-zero treated samples be *X*=(*x*_1_, *x*_2_,…, *x*_*M*_), *Y*=(*y*_1_, *y*_2_,…, *y*_*M*_). The objective of the CCA method is to combine equations (1) and (2) to find the maximum correlation between them.

(1) $$\begin{array}{c} x^{*}=\varphi_{x}^{T}x, \end{array}$$

(2) $$\begin{array}{c} y^{*}=\varphi_{y}^{T}y. \end{array}$$

The coefficients corresponding to the maximum value of the correlation are *φ*(**x**) and *φ*(**y**).

Let the covariance matrices of *X* and *Y* be *C*_*xx*_ and *C*_*yy*_, respectively, and the mutual covariance matrix be *C*_*xy*_.

(3) $$\begin{array}{c} \rho =\frac{\varphi_{x}^{T}C_{xy}\varphi_{y}}{\sqrt{\varphi_{x}^{T}C_{xx}\varphi_{x}\varphi_{y}^{T}C_{xy}\varphi_{y}}},\\ C_{xx}=\mathrm{cov}\left(x,x\right)=E\left(x^{2}\right)-{\left(E\left(x\right)\right)}^{2},\\ C_{yy}=\mathrm{cov}\left(y,y\right)=E\left(y^{2}\right)-{\left(E\left(y\right)\right)}^{2},\\ C_{xy}=\mathrm{cov}\left(x,y\right)=E\left(xy\right)-E\left(x\right)E\left(y\right), \end{array}$$

where *E*( ) denotes the expected solution.

(4) $$\begin{array}{c} \left\{\begin{array}{cc} \underset{\varphi_{x},\varphi_{y}}{\max} & \varphi_{x}^{T}C_{xy}\varphi_{y},\\ \text{s}.\text{t}. & \varphi_{x}^{T}C_{xx}\varphi_{x}=1,\;\varphi_{y}^{T}C_{yy}\varphi_{y}=1. \end{array}\right. \end{array}$$

The best *φ*(**x**) and *φ*(**y**) can be obtained by solving the above equation using the Lagrangian function method.

### 3.2. Nuclear Typical Correlation Analysis

**Figure 7:**
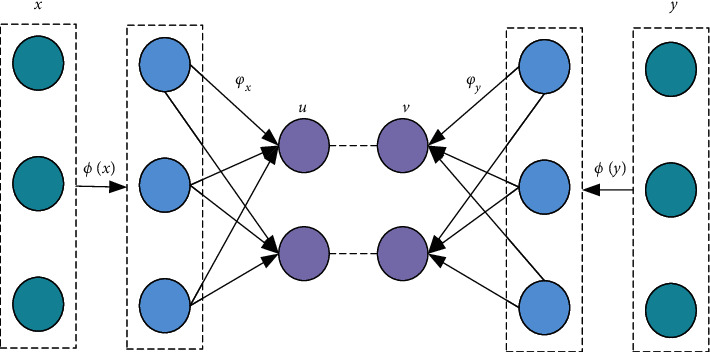
KCCA schematic diagram.

The kernel function is introduced on the basis of CCA to construct the kernel typical correlation analysis (KCCA) method, which better solves the correlation analysis between two different dimensional features [36], and the main structure is shown in Figure 7.

Let the mapping function *ϕ*(*x*) satisfy *K*(*x*, *y*)=〈*ϕ*(*x*), *ϕ*(*y*)〉, then *K*(*x*) is said to be the kernel function. The normalized samples (*X*=(*x*_1_, *x*_2_, ⋯, *x*_*N*_), *Y*=(*y*_1_, *y*_2_, ⋯, *y*_*N*_)) are mapped to the *ϕ* function, and then the correlation coefficients of the samples *X* and *Y* are solved according to equation (1).

(5) $$\begin{array}{c} \rho =\frac{\varphi_{x}^{T}K\left(x,y\right)\varphi_{y}}{\sqrt{\varphi_{x}^{T}K\left(x,x\right)\varphi_{x}\varphi_{y}^{T}K\left(y,y\right)\varphi_{y}}}. \end{array}$$

Then, we can get to the required constraint.

(6) $$\begin{array}{c} \left\{\begin{array}{cc} \underset{\varphi_{x},\varphi_{y}}{\max} & \varphi_{x}^{T}K\left(x,y\right)\varphi_{y},\\ \text{s}.\text{t}. & \varphi_{x}^{T}K\left(x,x\right)\varphi_{x}=1,\;\varphi_{y}^{T}K\left(x,y\right)\varphi_{y}=1. \end{array}\right. \end{array}$$

### 3.3. Support Vector Machines

Let the sample set (*x*_*i*_, *y*_*i*_) can be mapped by a nonlinear support vector machine to obtain the linear equation, *i*=1,2,…, *n*.

(7) $$\begin{array}{c} y_{i}\left(w^{T}x_{i}+b\right)-1\geq 0,\;i=1,2,\ldots ,n, \end{array}$$

where **w**^*T*^ is the weight matrix and *b* represents the bias.

The solution to equation (7) is converted to solving for the minimum of *ϕ*(**w**)=1/2‖**W**‖^2^=1/2(**w**^*T*^**w**). A Lagrangian transformation is performed to obtain the new solution equation.

(8) $$\begin{array}{c} L\left(w,b,a\right)=\frac{1}{2}\left(w^{T}w\right)-\sum_{i=1}^{n}a_{i}\left[y_{i}\left(w^{T}x_{i}+b\right)-1\right],\\ \text{s}.\text{.t}.\;\sum_{i=1}^{n}\alpha_{i}y_{i}=0, \end{array}$$

where *a*_*i*_ is the Lagrangian coefficient. Carry out bias derivative of equation (8) for **w** and *b*, respectively.

(9) $$\begin{array}{c} Q\left(a\right)=\sum_{i=1}^{n}a_{i}-\frac{1}{2}\sum a_{i}a_{j}y_{i}y_{j}\left(x_{i}x_{j}\right). \end{array}$$

Solve *Q*(**a**) to obtain the maximum value corresponding to **a**^*∗*^.

(10) $$\begin{array}{c} w^{*}=\sum_{i=1}^{n}y_{i}a_{i}^{*}x_{i}. \end{array}$$

Finally, the optimal SVM can be calculated as follows.

(11) $$\begin{array}{c} f\left(x\right)=\mathrm{sgn}\left({\left(w^{*}\right)}^{T}+b\right). \end{array}$$

## 4. KCCA-SVM-Based Security Monitoring Model for Historic Buildings

### 4.1. Monitoring Model Implementation Process

**Figure 8:**
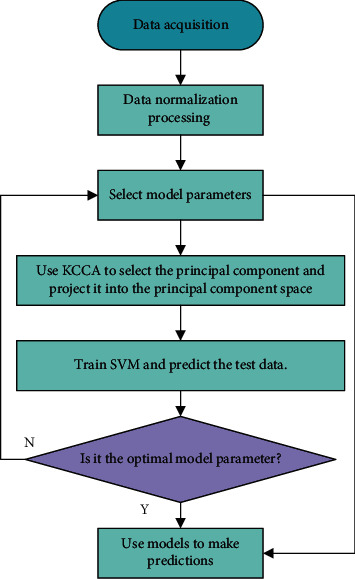
The flowchart of safety monitoring model based on KCCA-SVM.

In this paper, a KCCA-SVM-based safety monitoring model for historical buildings is proposed. Firstly, KCCA is used to preprocess the independent variables of the original data and extract the principal components (principal components represent the information synthesized by the independent variables according to different weights), so as to reduce the dimensionality of the data and eliminate the noise. During the training process, the kernel parameters can be adjusted to improve the fitting ability of the SVM. The best-fitting combination of parameters is selected as the model parameters.  Figure 8 shows the implementation process of the KCCA-SVM-based historical building safety monitoring model.

### 4.2. Validation of the Machine Learning Dataset

To verify the classification performance of KCCA-SVM, simulation tests were conducted using the commonly used UCI machine learning dataset, which is shown in Table 1.

#### 4.2.1. Influence of Different Kernel Functions

The selection of a suitable kernel function has a large impact on the feature extraction effect of KCCA, which directly affects the classification performance of SVM. Therefore, in this paper, different kernel functions are selected for KCCA analysis and then SVM classification. The KCCA-SVM recognition accuracies of different kernel functions are shown in Table 2.

From Table 2, KCCA has lower RMSE values for the ORL and PIE sets using Gaussian kernels, while KCCA has lower RMSE values for the Yale and AR sets using sigmoid kernels. Therefore, the Gaussian kernel is more accurate in the ORL and PIE sets, and the sigmoid kernel performs better in the Yale and AR sets.

#### 4.2.2. Influence of Different Variable Dimensions

To further validate the performance of KCCA-SVM, different variable dimensions were selected for KCCA analysis, followed by SVM identification. The recognition accuracies of the different dimensions are shown in Table 3.

From Table 3, the variable dimensionality has a significant impact on the accuracy of KCCA-SVM. At dimension 10, the KCCA-SVM recognition accuracy is the lowest. The accuracy of KCCA-SVM was higher when the number of dimensions was 20 and 25, and the two values were very close to each other. When the number of dimensions is small, the variables cannot contain important feature information, resulting in a low recognition accuracy. And when the number of dimensions was increased to 20, the accuracy did not appear to improve significantly when the number of dimensions continued to increase. This is mainly because after the dimensionality reaches 20, the selected variable features can already contain the sample attributes in a more comprehensive way. Therefore, even if the number of variable features is increased further, the accuracy does not increase significantly.

The simulation of recognition stability for different dimensions continues below, and the statistical results are shown in Table 4.

From Table 4, the RMSE values of the KCCA-SVM are decreasing as the number of dimensions increases, which indicates that an increase in the number of dimensions has a significant positive effect on stability. The RMSE values are still decreasing when the dimensionality is increased from 20 to 25, indicating that the full extraction of variable features is more beneficial to stability improvement. The dimensionality of variables can improve the stability of recognition, but it also brings a greater amount of recognition operations, which affects the recognition efficiency, so the dimensionality of image recognition should be selected according to the actual situation.

## 5. Project Examples

### 5.1. Historical Architectural Context

In this paper, the Shanghai Great World, which was rebuilt in 1924 on South Xizang Road in Huangpu District, is a reinforced concrete frame structure with an L-shaped plan. Shanghai World has a site area of 6537 m^2^ and a building area of 13580 m^2^. Its architectural style is mixed, including Western classical and Chinese traditional forms. Shanghai World is one of the representative buildings of modern entertainment architecture. Due to factors such as the excavation of the underground in the vicinity of Great World, safety issues have arisen in the structure of the building, such as tilting and cracking of the walls. In order to monitor the structural safety of Great World, a wireless sensor network was used to monitor the overall characteristics of the building structure.

In this paper, the manual monitoring data of vertical displacement from 8 July 2010 to 8 July 2019 were selected as the research samples to build a safety monitoring model based on KCCA-SVM. A total of 205 groups were sampled, with the first 190 groups used as training samples and the last 15 as testing samples. The main factors of settlement affecting building safety include 3 aspects, namely, temperature, pressure, and time duration, and each factor consists of a number of vectors. Therefore, a total of 14 factors were selected as the initial input vectors, including 4 temperature factors, 8 pressure factors, and 2 aging factors.

### 5.2. Results of the Safety State Assessment

**Figure 9:**
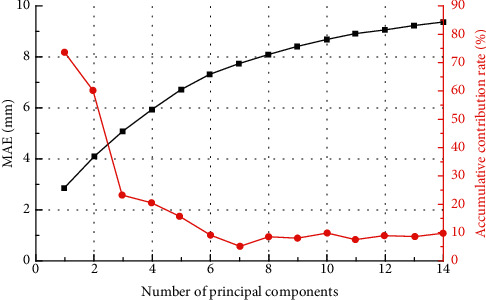
The relationship between the number of principal components extracted of KCCA and the monitoring model.

In the security monitoring model, the kernel matrix of KCCA (190 × 190) is obtained from the input sample matrix (190 × 14). The number of principal components extracted by KCCA may be greater than the number of independent variables in the initial sample (14). The highest number of samples was 190. The exact number of principal components to be extracted should be determined through analytical studies. When the kernel parameter is *g*=25.6 and *g*′=5.76, the SVM outperforms the SVM with other kernel parameters, regardless of the number of principal components extracted. Therefore, the kernel parameter is fixed to *g*=25.6 and *g*′ = 5.76. The relationship between the number of principal components extracted by KCCA and the computational results of the security monitoring model is investigated, as shown in Table 5 and Figure 9.

When the SVM kernel parameters are fixed, the mean absolute error (MAE) of the KCCA-SVM-based security monitoring model first tends to decrease as the number of principal components extracted by KCCA increases. When the number of principal components is 7, the MAE reaches a minimum and then increases slightly when the number of principal components increases to 8. Subsequently, as the number of extracted principal components increases, the MAE fluctuates slightly but gradually plateaus. Therefore, the number of principal components in the security monitoring model is not as large as possible. When the number of extracted principal components is 7, the prediction accuracy of SVM has reached a high level and there is no need to extract more principal components as input independent variables. Further increase in the number of principal components will introduce more noise and affect the prediction accuracy of the model.

**Figure 10:**
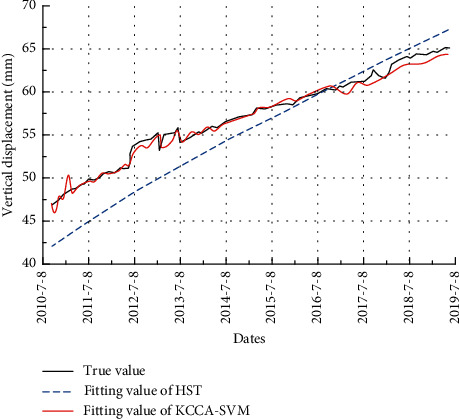
The settlement fitting value of HST and KCCA-SVM model.

In the safety monitoring model, a reasonable number of principal components are extracted using KCCA, which can achieve the purpose of eliminating data noise, reducing data dimensionality and improving model prediction accuracy. The number of principal components extracted for the safety monitoring model is determined to be 7, and the corresponding cumulative contribution rate is 69.3%. Compared with the 14 input vectors of the original data, the data dimensionality reduction is very obvious.  Figure 10 shows a comparison of the fitted values of the training data for the conventional statistical regression model (HST) and the KCCA-SVM model.

It can be seen that the fitting effect of the conventional HST model deviates significantly with the measured settlement data as a benchmark. The KCCA-SVM-based safety monitoring model, on the other hand, has a significantly better fit. The good fit of the model provides a basis for the subsequent accurate prediction of settlement of historic buildings.

In order to better represent the fitting ability and generalization ability of the KCCA-SVM-based security monitoring model, this paper has built HST, SVM, RVM, PCA-SVM, PCA-RVM, and KCCA-SVM models simultaneously. These models use the same training samples and make predictions on the same test data. The prediction effectiveness was evaluated using the maximum relative error, mean relative error, and mean absolute error metrics. A comparison of the prediction accuracy is shown in Table 6.

**Figure 11:**
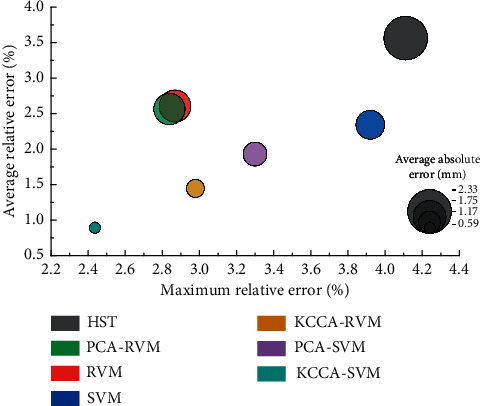
Comparison of errors of different settlement prediction models.

We can see that the traditional HST model has a significant error. The prediction accuracy of machine learning algorithms SVM and RVM is significantly higher than that of the HST model. Compared to not using the preprocessing algorithm, the prediction accuracy was slightly improved by using the PCA model to extract the principal components from the input data and then using the SVM and RVM models to make predictions. This indicates that PCA has some noise removal effect. The prediction accuracy of both the KCCA-SVM and KCCA-RVM models improved substantially after using the KCCA model for nonlinear principal component extraction of the input data. The main reason for this is that KCCA has a better handling of the nonlinear features present in the original subsidence data. Among the various compared algorithms, SVM is significantly faster than RVM, due to the sparsity of the results of SVM. For a more visual display, a bubble diagram was used to represent Table 6, as shown in Figure 11.

In summary, the KCCA-RVM-based historical building safety state prediction model has the advantages of reduced data dimensionality, noise elimination, fast calculation speed, and high prediction accuracy.

## 6. Conclusion

This paper attempts to combine two approaches to build a KCCA-SVM-based security monitoring model for historic buildings. Firstly, wireless sensor networks are applied to the security monitoring of historic buildings in order to improve the automation of monitoring. Secondly, KCCA technique is used for feature correlation analysis to reduce the dimensionality of a large amount of nonlinear data. SVM is then used to take full advantage of its strengths in handling nonlinear and high-dimensional data to predict multiple physical quantities of historic buildings, improving the accuracy of security state assessment. Test results on commonly used machine learning datasets and engineering examples show that the KCCA-SVM model can accurately predict physical quantities such as relative structural displacements of historic buildings.

## Figures and Tables

**Table 1:** UCI machine learning dataset.

Sample set	Number of samples	Number of attributes
ORL	400	30
Yale	400	32
AR	400	28
PIE	400	30

**Table 2:** KCCA-SVM recognition accuracy of different kernel functions.

Datasets	Kernel functions	RMSE		
		Minimum value	Average value	Maximum value
ORL	Gaussian kernel	8.322*e* − 2	8.581*e* − 2	8.676*e* − 2
	Sigmoid nucleus	8.693*e* − 2	8.825*e* − 2	8.991*e* − 2
	Polynomial kernel	9.592*e* − 2	9.674*e* − 2	1.201*e* − 1

Yale	Gaussian kernel	1.044*e* − 1	1.124*e* − 1	1.265*e* − 1
	Sigmoid nucleus	9.817*e* − 2	9.924*e* − 2	1.063*e* − 1
	Polynomial kernel	1.249*e* − 1	1.535*e* − 1	1.922*e* − 1

AR	Gaussian kernel	8.465*e* − 2	8.573*e* − 2	8.834*e* − 2
	Sigmoid nucleus	7.835*e* − 2	7.902*e* − 2	8.064*e* − 2
	Polynomial kernel	9.437*e* − 2	9.611*e* − 2	9.947*e* − 2

PIE	Gaussian kernel	7.916*e* − 2	8.111*e* − 2	8.259*e* − 2
	Sigmoid nucleus	8.146*e* − 2	8.297*e* − 2	8.481*e* − 2
	Polynomial kernel	1.098*e* − 1	1.244*e* − 1	1.367*e* − 1

**Table 3:** Recognition accuracy of KCCA-SVM with different dimensions.

Datasets	Dimension	Accuracy		
		Minimum value	Average value	Maximum value
ORL	10	0.728	0.735	0.751
	15	0.841	0.857	0.866
	20	0.921	0.922	0.923
	25	0.922	0.922	0.923

Yale	10	0.769	0.782	0.791
	15	0.838	0.849	0.858
	20	0.902	0.903	0.906
	25	0.906	0.906	0.906

AR	10	0.842	0.855	0.861
	15	0.882	0.897	0.908
	20	0.943	0.952	0.958
	25	0.950	0.956	0.958

PIE	10	0.812	0.821	0.833
	15	0.863	0.872	0.884
	20	0.887	0.896	0.901
	25	0.907	0.913	0.916

**Table 4:** Recognition accuracy of RMSE of KCCA-SVM with different dimensions.

Datasets	Dimension	RMSE		
		Minimum value	Average value	Maximum value
ORL	10	8.715*e* − 2	8.927*e* − 2	9.142*e* − 2
	15	8.646*e* − 2	8.893*e* − 2	9.013*e* − 2
	20	8.321*e* − 2	8.581*e* − 2	8.676*e* − 2
	25	8.136*e* − 2	8.327*e* − 2	8.435*e* − 2

Yale	10	1.253*e* − 1	1.277*e* − 1	1.294*e* − 1
	15	1.175*e* − 1	1.263*e* − 1	1.381*e* − 1
	20	9.817*e* − 2	9.924*e* − 2	1.063*e* − 1
	25	9.656*e* − 2	9.721*e* − 2	9.826*e* − 2

AR	10	8.486*e* − 2	8.579*e* − 2	8.721*e* − 2
	15	8.101*e* − 2	8.243*e* − 2	8.375*e* − 2
	20	7.836*e* − 2	7.903*e* − 2	8.064*e* − 2
	25	7.811*e* − 2	7.885*e* − 2	7.975*e* − 2

PIE	10	8.721*e* − 2	8.835*e* − 2	8.907*e* − 2
	15	8.367*e* − 2	8.472*e* − 2	8.563*e* − 2
	20	7.916*e* − 2	8.112*e* − 2	8.259*e* − 2
	25	7.875*e* − 2	8.002*e* − 2	8.157*e* − 2

**Table 5:** The relationship between the number of principal components extracted of KCCA and the monitoring model.

Number of principal components	Cumulative contribution (%)	Average absolute error (mm)
1	25.9	8.14
2	36.78	6.68
3	45.27	2.62
4	52.87	2.31
5	60.1	1.81
7	65.32	0.94
10	69.27	0.59
11	72.57	0.96
12	75.52	0.88
13	77.68	1.11
14	79.74	0.83

**Table 6:** Error comparison of different settlement prediction models based on training data.

Models	Maximum relative error (%)	Average relative error (%)	Average absolute error (mm)
HST	4.11	3.55	2.3256
RVM	2.87	2.6	1.7025
SVM	3.92	2.34	1.5357
PCA-RVM	2.84	2.56	1.6789
PCA-SVM	3.3	1.93	1.2641
KCCA-RVM	2.98	1.45	0.953
KCCA-SVM	2.44	0.9	0.5915

## Data Availability

The experimental data used to support the findings of this study are available from the corresponding author upon request.
